# Multicomponent Analysis of Junctional Movements Regulated by Myosin II Isoforms at the Epithelial Zonula Adherens

**DOI:** 10.1371/journal.pone.0022458

**Published:** 2011-07-22

**Authors:** Michael Smutny, Selwin K. Wu, Guillermo A. Gomez, Sabine Mangold, Alpha S. Yap, Nicholas A. Hamilton

**Affiliations:** 1 Division of Molecular Cell Biology, Institute for Molecular Bioscience, The University of Queensland, St. Lucia, Brisbane, Queensland, Australia; 2 Division of Genomics and Computational Biology, Institute for Molecular Bioscience, The University of Queensland, St. Lucia, Brisbane, Queensland, Australia; Northwestern University Feinberg School of Medicine, United States of America

## Abstract

The zonula adherens (ZA) of epithelial cells is a site of cell-cell adhesion where cellular forces are exerted and resisted. Increasing evidence indicates that E-cadherin adhesion molecules at the ZA serve to sense force applied on the junctions and coordinate cytoskeletal responses to those forces. Efforts to understand the role that cadherins play in mechanotransduction have been limited by the lack of assays to measure the impact of forces on the ZA. In this study we used 4D imaging of GFP-tagged E-cadherin to analyse the movement of the ZA. Junctions in confluent epithelial monolayers displayed prominent movements oriented orthogonal (perpendicular) to the ZA itself. Two components were identified in these movements: a relatively slow unidirectional (translational) component that could be readily fitted by least-squares regression analysis, upon which were superimposed more rapid oscillatory movements. Myosin IIB was a dominant factor responsible for driving the unilateral translational movements. In contrast, frequency spectrum analysis revealed that depletion of Myosin IIA increased the power of the oscillatory movements. This implies that Myosin IIA may serve to dampen oscillatory movements of the ZA. This extends our recent analysis of Myosin II at the ZA to demonstrate that Myosin IIA and Myosin IIB make distinct contributions to junctional movement at the ZA.

## Introduction

Cell-cell contacts experience force: they are sites where force is generated, resisted and sensed [1], [2], [3]. This has been clearly demonstrated during development, notably in Drosophila embryos, where a variety of tensile forces act upon cell-cell contacts to mediate morphogenetic movements and cellular patterning [4], [5], [6], [7], [8], [9]. The impact of forces at junctions, however, has been less well-characterized in vertebrate, including mammalian, systems.

Cell-cell adhesion receptors potentially play central roles in many aspects of mechanobiology at intercellular junctions. In epithelial cells, E-cadherin is a major contributor to cell-cell adhesion [10], where it accumulates in the zonula adherens (ZA), a belt-like structure found at the apical interface between cells [11], as well as in clusters throughout the lateral contacts. E-cadherin is predicted to mechanically couple cell surfaces to one another, thereby serving as a key mechanical element that experiences force between cells. Indeed, recent evidence implicates cadherins in mechanosensing at cell-cell contacts [3], [12], [13]. E-cadherin can also coordinate the cytoskeletal reponse to those forces, by recruiting a range of actin regulators [14], either through protein-protein interactions and/or adhesion-activated signaling pathways [15]. These include tension sensitive actin-based anchors, such as Myosin VI [16], [17], which may be involved in cellular resistance to force and proteins such as α-catenin that can participate in sensing tension applied to contacts [18].

The major contractile force-generator found at cell-cell contacts is the actin-binding motor, non-muscle Myosin II, which contributes to junctional tension in Drosophila [7], [19], [20] and supports ZA integrity [21], [22] and tissue integrity in mammalian epithelial systems [23]. Of note, however, whereas Drosophila have a single Myosin II gene, three Myosin II isoforms are found in mammals and it is increasingly evident that these can have quite different biophysical and biological effects [24]. We recently reported that both Myosin IIA and Myosin IIB are found contiguous with the ZA and are necessary for ZA integrity [22]. However, these Myosin II isoforms contributed to junctional integrity by different mechanisms. Myosin IIA appeared to function principally as a regulator of cortical organization, promoting cadherin clustering and its concentration into the ring-like structure of the ZA. Myosin IIB, in contrast, supported the integrity of the apical ring of actin filaments that is found in association with the ZA. Taken with evidence that their junctional localization responded to different upstream signals, this suggested that these Myosin II isoforms identified distinct functional modules that acted upon the ZA.

In this report, we explore further the potential differential impacts of Myosin IIA and Myosin IIB on the ZA. We have analysed the movement of junctional E-cadherin as a parameter to potentially report the play of forces upon junctions and developed protocols to quantitate patterns of junctional movement in 4D movies. We now report that multiple patterns of junctional movement are found at contacts between cells in confluent epithelial monolayers. Focusing on movements oriented perpendicular (orthogonal) to the ZA itself we find that the ZA displays two patterns of movement: a slow pattern of unidirectional (translational) movement and much faster patterns of oscillation superimposed upon the overall trend of the translational movement. Whereas Myosin IIB is principally responsible for driving the unidirectional translational movement, Myosin IIA serves to dampen the oscillatory pattern.

## Results

### A model system to analyse movement dynamics of E-cadherin at the ZA

Our first aim was to develop a model system to study the dynamics of E-cadherin movement at epithelial cell-cell contacts. Initial experiments suggested that simple overexpression of EGFP-tagged E-cadherin might be associated with gain-of-function effects when observed by live cell imaging (data not shown). Hence we chose to express EGFP-tagged E-cadherin in cells where endogenous E-cadherin was depleted by RNAi. We constructed a lentivirus vector [25], [26] that allows simultaneous expression of an shRNA specific for human E-cadherin driven from a Pol III U6 promoter and also full length mouse E-cadherin-EGFP (E-Cad-GFP) from a LTR promoter.

**Figure 1:**
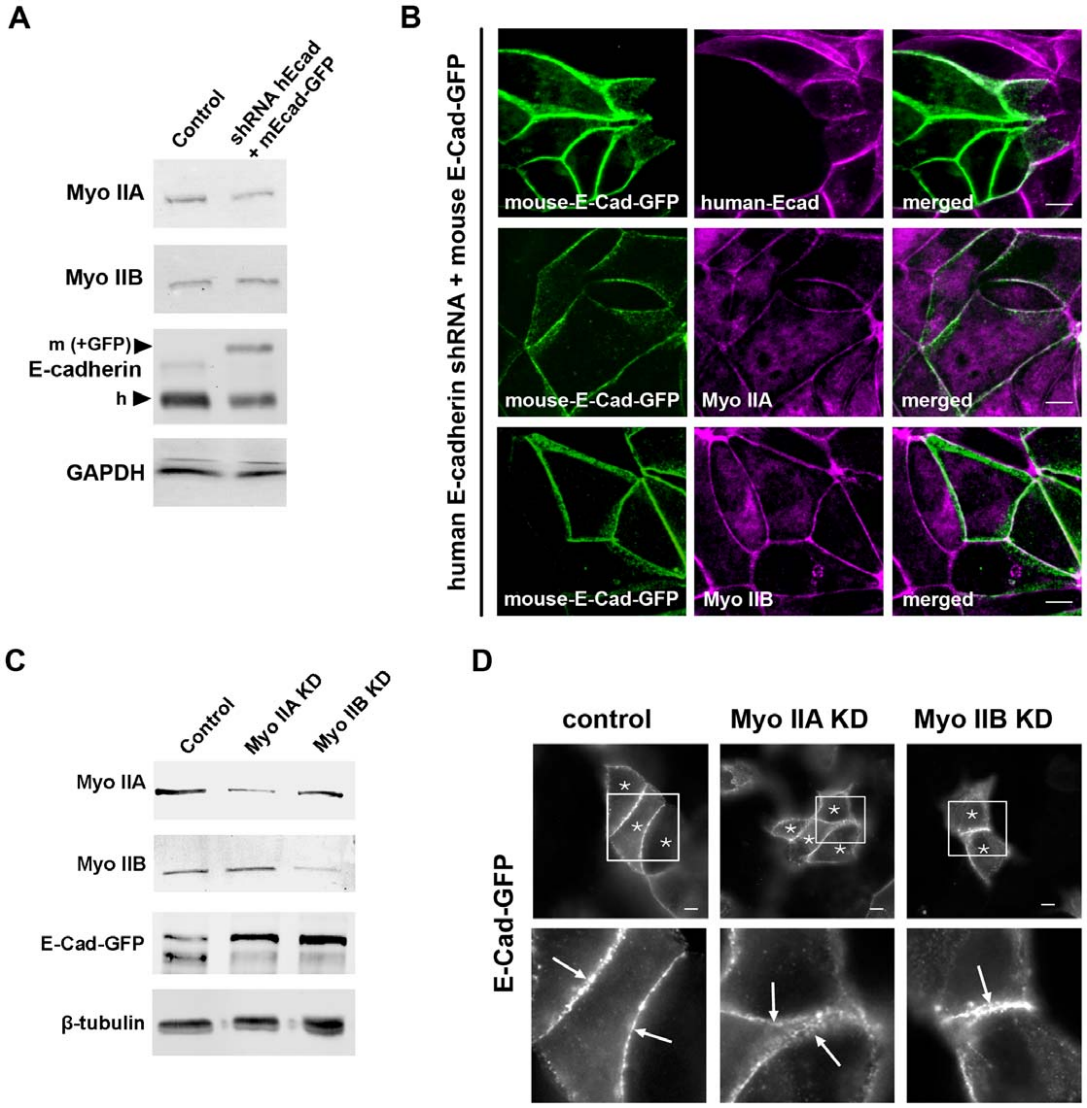
Knockdown of human E-cadherin in MCF-7 cells and reconstitution with mouse EGFP-E-cadherin. (A) E-cadherin KD/E-cad-GFP reconstituted cell line: MCF-7 cells were infected with lentivirus bearing both shRNA directed against human E-cadherin and full-length mouse E-cadherin fused to EGFP. Immunoblots were probed for E-cadherin, Myosin IIA and IIB, and GAPDH. Protein levels of endogenous human E-cadherin (h) and expressed mouse Ecad-GFP (m) were recognized by the same E-cadherin antibody against the cytoplasmic tail. (B) E-cadherin KD/reconstituted MCF-7 cells were fixed and immunostained for human E-cadherin (magenta) to confirm knockdown and for EGFP (green) to visualize exogenous expression of E-cad-GFP. Cells were also costained for E-cad-GFP (green) and Myosin IIA (magenta) or Myosin IIB (magenta). Images shown are representative confocal sections from the apical junctions. (C) E-cadherin KD/reconstituted MCF-7 cells were infected with lentiviral shRNA directed against either Myosin IIA or Myosin IIB or with control empty virus expressing mCherry alone. Immunoblots show knockdown levels of Myosin IIA and IIB and expression levels of E-Cad-GFP. Tubulin was used as a loading control. (D) E-cadherin KD/reconstituted MCF-7 cells were infected with lentiviral shRNA directed against either Myosin IIA or Myosin IIB or with control empty virus expressing mCherry alone. Transduced cells express mCherry and are marked with asterisks. Fixed cells were immunostained for EGFP-E-cadherin and morphology of exogenously expressed E-cadherin in knockdown and control cells is shown at apical junctions. Arrows in enlarged images indicate accumulation of E-Cad-GFP at the ZA of cell-cell contacts. Scale bars = 10 µm.

Immunoblot analysis with an E-cadherin antibody that recognizes both endogenous human E-cadherin and the mouse E-cadherin transgene demonstrated depletion of endogenous cadherin (Fig. 1A). Comparison of polypeptide levels by immunoblotting for E-cadherin suggested that overall the combination of residual endogenous E-cadherin and the transgene was similar to the endogenous levels of E-cadherin in control cells. However, immunofluorescence analysis suggested that the apparently partial degree of knock-down detected by immunoblotting is likely due to cellular heterogeneity (Fig. 1B). Of note, mouse E-Cad-GFP was consistently expressed in cells depleted of endogenous E-cadherin, demonstrating effectively “replacement” of endogenous E-cadherin with the GFP-tagged transgene.

In confluent monolayers, E-Cad-GFP was found throughout the lateral interfaces between cells where it concentrated in an apical ring-like structure, consistent with the ZA, as well as in puncta found throughout the lateral surface, below the apical ZA ring (Fig 1B). Indeed, the ring-like organization of the ZA appears to reflect the concentration of cadherin clusters at the apico-lateral interface between cells. Furthermore, E-Cad-GFP at the ZA was contiguous with apical junctional Myosin IIA and Myosin IIB (Fig. 1B), as is also seen with endogenous E-cadherin [22]. Total cellular levels of these Myosin II isoforms were unchanged in the E-cad-GFP reconstitution cells (Fig. 1A).

In order to examine the potential impact of these Myosin II isoforms on junctional movement of E-cadherin in the ZA, we then depleted each Myosin II isoform in MCF7 cells expressing E-Cad-GFP using the isoform-specific lentiviral shRNA approach that we reported previously [22]. We used Myosin II KD virus that co-expressed soluble mCherry as an infection marker to select for cells with moderate to high knockdown levels. Knockdown of either Myosin IIA or IIB in E-Cad-GFP reconstituted cells was achieved without perturbing the expression levels of the other Myosin II isoform or of E-Cad-GFP (Fig 1C). Immunofluorescence confirmed loss of the appropriate isoform from the junctions of Myosin IIA or IIB KD cells (Fig S1). While E-Cad-GFP continued to localize at cell-cell contacts despite Myosin II KD (Fig S1), E-Cad-GFP did not accumulate into the ZA as intensely in Myosin IIA KD cells as it did in control cells and there was fragmentation of the ZA in Myosin IIB KD cells (Fig. 1D), consistent with what we had earlier observed for endogenous E-cadherin [22].

### Myosin IIB is necessary for sustained orthogonal movement of E-cadherin at the ZA

**Figure 2:**
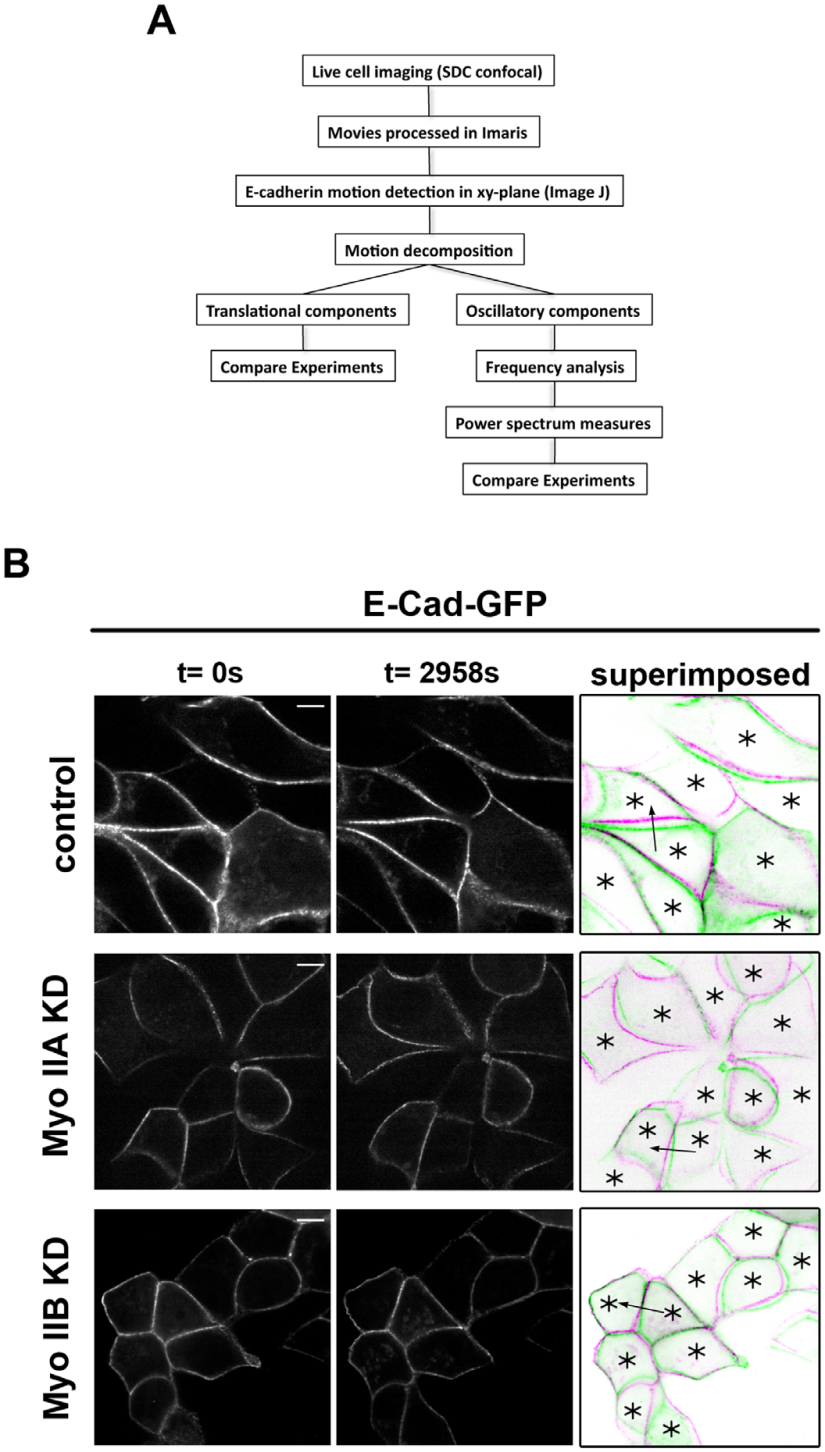
Visualization and characterization of orthogonal E-cadherin movements in live cells. (A) The workflow summarizes the experimental setup and data analysis. (B) E-cadherin dynamics at cell-cell contacts were visualized using 4D live cell imaging and representative z-sections were taken from movies at t = 0 s and t = 2958 s. Superimposed images show movements of E-cadherin at contacts at t = 0 s (green) and t = 2958 s (magenta); asterisks mark transduced cells (mCherry marker). Arrows indicate cell-cell contacts that were used as in Figure 3 for representative analysis. Scale bars = 10 µm.

We then utilized 4D live cell imaging to study the dynamic movements of junctional E-cadherin in cell monolayers. This involved recording full-depth image stacks, but for this analysis we focused on the behaviour of E-Cad-GFP in the apical ZA ring. Visual inspection of movies captured over ∼50 min showed complex patterns of movement at the ZA. In addition to motion of local concentrations of E-cadherin along the ZA (“lateral” motions) we noticed that the ZA itself appeared to undergo movements directed perpendicular or orthogonal to the junction itself (Movie S1), an orientation potentially informative about forces exerted on the ZA. We therefore elected to focus on analysing these orthogonal movements and developed an analysis pipeline to quantitatively characterize these orthogonal movements, as outlined Fig. 2A.

As a first approach to developing a visual representation of these orthogonal movements, we superimposed start and end frames of individual movies upon one another (Fig. 2B). We predicted that if junctions did not move, then this would yield precise superimposition of ZA patterns, whereas orthogonal movement would cause translocation of the ZA pattern at the end of the movie. Indeed, control cells showed displacement of E-Cad-GFP consistent with orthogonal movement, but little apparent translocation of the cell bodies themselves. Interestingly, whereas movies of Myosin IIA KD cells also showed displacement, this was less apparent in Myosin IIB KD cells, suggesting that Myosin IIB cells might have reduced orthogonal movement of the ZA. To get an indication of movements throughout the movies, we also superimposed all frames of movies upon one another (Fig S2). Whereas both control and Myosin IIA KD cells showed quite broad dispersion of fluorescence, Myosin IIB KD cells showed a much tighter distribution of fluorescence, consistent with more limited movement.

**Figure 3:**
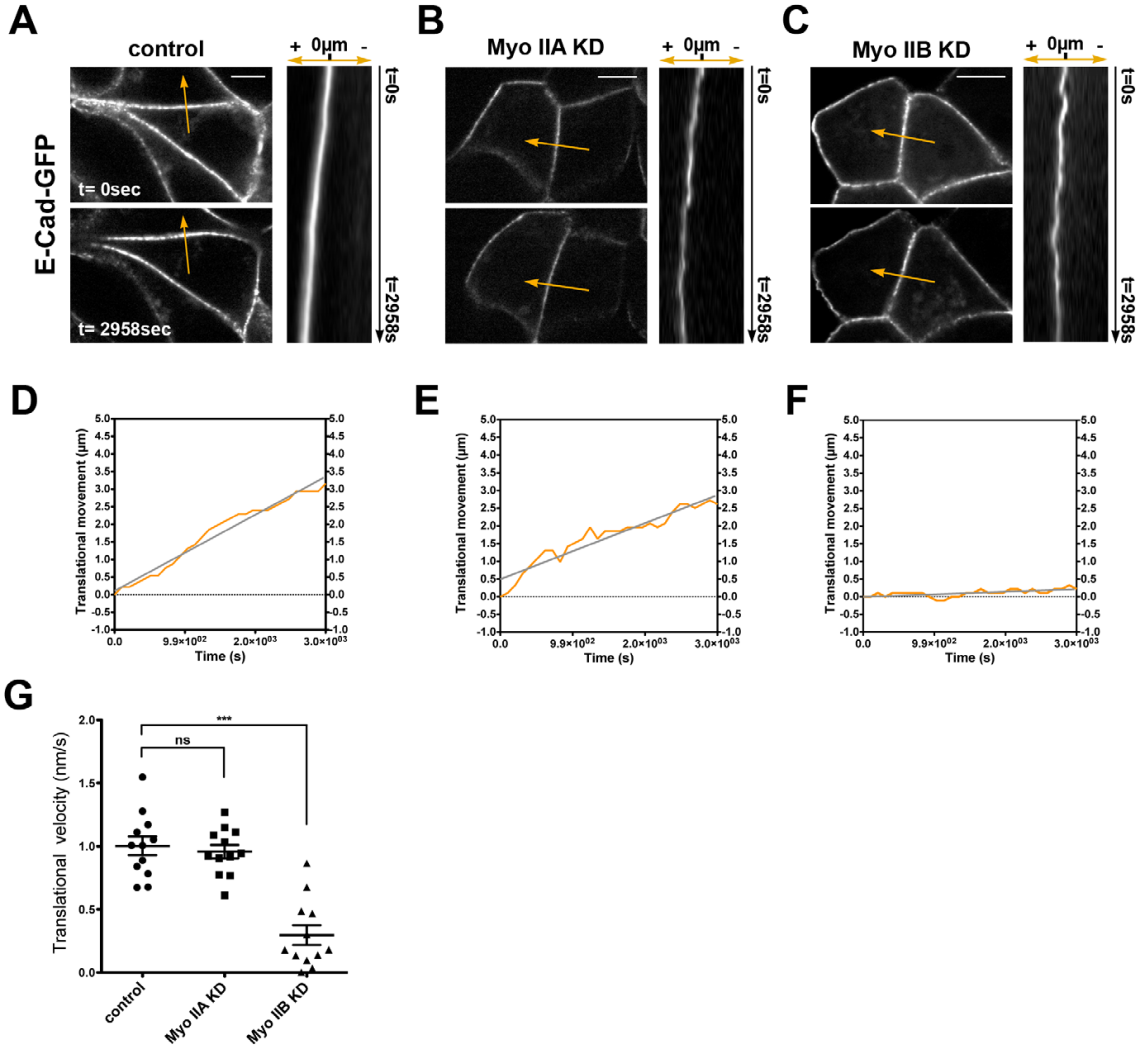
Translational movement of E-cadherin at the ZA requires Myosin IIB. Images are enlarged representatives of KD and control cells taken from movies (Fig 2B, Movie S2, S3, S4) and were used as examples to illustrate differences in movement of junctional E-cadherin in Myosin II KD cells. (A–C). Translational movement of E-cadherin (orange line) in control (**A**), Myosin IIA KD (**B**) and Myosin IIB KD (**C**) cells was measured from single cell-cell contacts. Kymographs show movement of E-cadherin over 2958 s in the xy-plane along the measured line scan (line with arrow as direction of movement). (D–F). The translational movement of E-cadherin at contact (orange line) was plotted in a graph corresponding to covered distance over time (µm/s) and a slope of the movement as best-fit line (grey) was calculated for control (D), Myosin IIA KD (E) and Myosin IIB KD (F). (G) Translational velocities of E-cadherin were calculated from the corresponding slopes; whisker plots show distribution of velocities for control, Myosin IIA KD and Myosin IIB KD cells in nm/s. Data are mean_s_ ± SEM (***p<0.001, ANOVA with Dunnet's test, n = 12 pooled from 4 independent movies). Scale bars = 10 µm.

We then used a kymographic approach to quantitatively analyze orthogonal junctional movements from movies of control and Myosin IIA and IIB knockdown cells (Fig. 3A–C, Movie S2, S3, S4). Randomly selected points along the ZA were selected for analysis and kymographs at these points extracted from the movies. When graphed as shown for individual measurements in Fig. 3D–F, control and Myosin IIA KD cells showed a consistent overall pattern of unidirectional displacement, which we refer to as “translational” movement of the ZA. Interestingly, translational movement seems to be drastically reduced in Myosin IIB KD cells (Fig. 3F). This translational movement could be fitted to a least-squares best-fit line and the translational velocity of a contact could be obtained from the slope of the best-fit line.

To describe changes of translational velocities in Myosin II depleted cells we compared least-squares best-fit regression lines extracted from control, Myosin IIA KD and Myosin IIB KD cells (Fig. 3G, Fig. S3). Control cells displayed a translational velocity of 1.003 (±0.07376) nm/sec that was not significantly affected by depletion of Myosin IIA (0.9575 0.05332 nm/s). In contrast, translational movement was significantly reduced in the Myosin IIB KD cells (0.2963±0.07821 nm/s). Overall, this analysis revealed that the ZA in confluent MCF7 monolayers displays a pattern of unidirectional translational movement that is substantially driven by the action of Myosin IIB, but does not appear to require Myosin IIA.

### Myosin IIA dampens rapid oscillatory movements of the ZA

In addition to the overall unidirectional pattern of translational movement, we noticed that junctions also displayed a faster pattern of back-and-forth movement around the line of translational movement (Fig. 3A–F). That is, junctions appeared to show a pattern of oscillatory movement superimposed on the unidirectional translational movement. We extracted this oscillatory component of junctional movement by subtracting the best-fit line of the translational component from the raw position data and plotting the motion along the time axis (µm/s). Representative de-trended oscillatory patterns are illustrated in Fig. S4. Often the detrended oscillatory patterns in control cells appeared sinusoidal with a clear period (Fig, S4A). To quantitatively analyse these sinusoidal features, we performed Fast Fourier Transform (FFT) analysis on the detrended data (see Methods). The FFT represents the signal uniquely as a sum of sinusoidal signals of differing frequencies. The square of the amplitude of each sinusoidal signal then corresponded to the power at that frequency and gives a measure of the strength of the oscillation at that frequency. Thus FFT analysis allowed us to decompose the oscillatory behaviour into the contribution of component frequencies.

**Figure 4:**
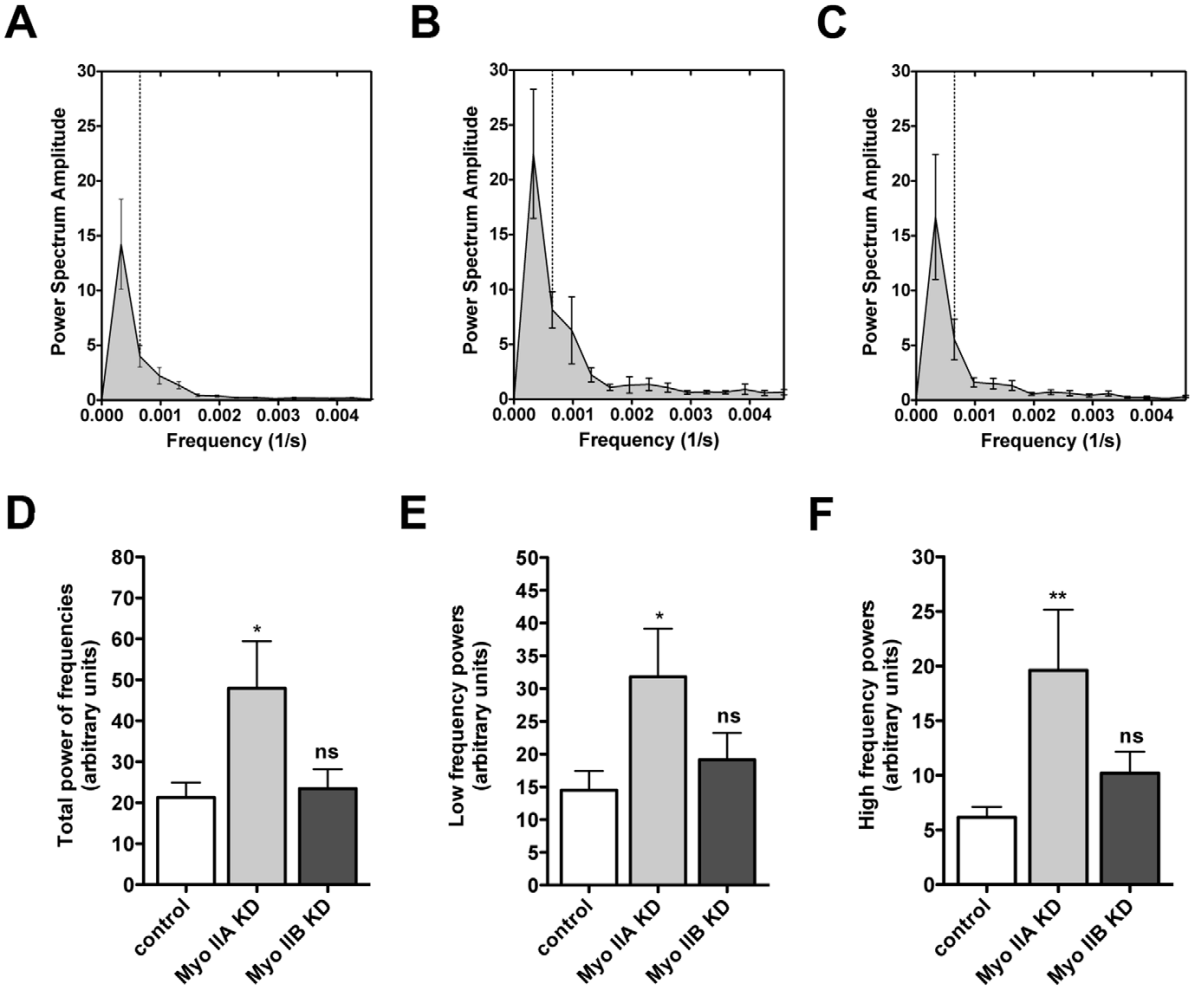
Controlled oscillatory motion of E-cadherin requires Myosin IIA. (A–C) Oscillatory frequencies of E-cadherin were analyzed by FFT and power spectrums were plotted for control (A), Myosin IIA KD (B) and Myosin IIB KD (C) cells. All frequencies are given in 1/s and the y-axis represents the power spectrum amplitudes for corresponding frequencies. Dashed lines mark the border between low and high frequency powers. (D–E) Differences in total power (D), low frequency power (E) and high frequency power (F) were calculated and plotted as bar graphs. Data are means ± SEM (*p<0.05, **p<0.01, ANOVA with Dunnett's test; n = 25–32 pooled from 4 separate movies).

In order to compare these oscillatory behaviours across experiments we then performed a FFT of the oscillatory component time series of junctions in the three cell lines (Fig. 4). The generated power spectrums (Fig 4A–C) show the relative strength of the signal at each discrete frequency of the FFT, thereby providing a measure of the amplitude of junctional oscillation at each frequency. The total powers (sum of powers at each frequency) are shown for each experiment type in Figure 4D.

In control cells, the power spectrum curves (Fig. 4A) showed a peak in the low frequency range (0.000327 s^−1^), which tapered off rapidly at higher frequencies. This indicated that control junctions displayed predominantly a pattern of relatively slow, sinusoidal oscillations that occurred around the overall unidirectional trend of translational movement. To determine if these overall patterns were influenced by the rate of image sampling, we imaged control cells at twice the original frame rate (i.e. frame intervals of 51 sec rather than 102 sec) for a total of 60 frames. This allowed power spectra to be computed up to 0.00948 Hz, i.e. twice the previous highest frequency, potentially allowing us to identify more prominent high-frequency oscillatory activity. However, no stronger signals were observed at the higher frequencies and the power spectra profiles were again similar to those shown in Fig 4A–C (data not shown).

In contrast, Myosin IIA KD cells displayed a significant increase in total power in the FFT analysis (Fig. 4D), indicating that the overall amplitude of oscillatory movement was increased compared with controls, despite the fact that translational movement was not different (Fig. 3G). There were clear trends for the power spectrum to be increased both in the low and high frequency ranges, while high frequency powers were increased twice as much as low frequency powers (Fig. 4E–F). The increased power at the low frequencies similar to those that were dominant in controls, suggested that the amplitude of excursion was increased for the lower frequency (sinusoidal) oscillations. The increased power at higher frequency further suggested that the oscillatory behaviour was more irregular in Myosin IIA KD cells than in controls. This implied that oscillations in Myosin IIA KD cells displayed both wider excursions and less smooth patterns. In contrast, no statistically significant increase in total power (Fig. 4D), power at low frequencies (Fig. 4E) or power at higher frequencies (Fig. 4F) was observed in Myosin IIB KD cells compared with controls.

## Discussion

Our analysis demonstrates that the ZA in confluent cultured epithelial cells undergoes orthogonal movements, oriented approximately perpendicular to the plane of the junction. Over the time course of our studies (∼50 min) these orthogonal movements comprise two discernible components: an underlying essentially unidirectional translational component, upon which are superimposed sinusoidal oscillatory movements. The two Myosin II isoforms that we have identified at the ZA appear to make distinct contributions to these different patterns of movement. Myosin IIB is critical to drive the underlying translational movement, as this component is significantly reduced in Myosin IIB KD cells, but unaffected by depleting Myosin IIA. In contrast, Myosin IIA appears to influence the pattern of oscillatory components. Whereas control junctions predominantly showed relatively low frequency oscillations around the overall translational trend, Myosin IIA KD cells were distinguished by higher amplitude oscillations and increased high frequency activity, indicating that the sinusoidal activity was displaying wider excursions and showing greater irregularity. This extends our recent analysis [22] to further demonstrate the capacity of these Myosin isoforms to make functionally distinct contributions to the regulation of ZA behaviour.

How, then, might IIB influence translational movement? One possibility is that Myosin IIB at the ZA may generate tension at the junctions. Myosin IIB is a high-duty ratio motor, which spends a high proportion of its mechanochemical cycle bound to actin filaments [24], [27]. This property is well-suited to generating tonic contractility [27]. Small imbalances in junctional tension on either side of a ZA would then be predicted to lead to relatively slow unidirectional movement of the junction, such as the translational behaviour that we observed. In contrast, Myosin IIA has a lower duty ratio than does Myosin IIB [27], making it less suited to tonic contractility, consistent with our observation that Myosin IIA depletion did not affect the translational movement of the ZA. We suspect that in cultured epithelial monolayers any differences in junctional tension are likely to be stochastic. However, planar polarized differences in Myosin IIB contractility at junctions would be a potential mechanism to co-ordinately alter cell shape in epithelial populations.

Whereas Myosin IIB appeared to contribute positively to orthogonal junctional movement, Myosin IIA appeared to principally exert an inhibitory effect to dampen the oscillatory movements that were identified by Fast Fourier Transform analysis. This dampening effect contains two elements. The increased power spectrum at low oscillatory frequencies seen in Myosin IIA KD cells implies that this motor isoform may limit the amplitude of excursion in the low frequency (sinusoidal) oscillations that are apparent in control cells. Additionally, however, the increased power in the high frequency range indicates that oscillations were more irregular in the Myosin IIA KD cells. Thus, although Myosin II has been implicated in driving various cellular oscillatory behaviours [28], [29], [30], in our system Myosin IIA appeared to principally limit the amplitude of sinusoidal oscillations at the ZA and ensure that they remain smooth and regular.

We do not yet know how Myosin IIA may have exerted this dampening effect on orthogonal ZA movements. We recently demonstrated that Myosin IIA supports the lateral clustering of E-cadherin and its accumulation in dense continuous clusters forming a ring-like pattern of the ZA [22]. Lateral clustering promotes strengthening of homophilic cadherin adhesion [31] and the amplification of adhesion might provide resistance to counterbalance the action of orthogonally-directed force generators. Additionally, the organization of cadherins into the ZA could distribute the generation of force, thereby dampening the amplitude of oscillations.

Our current findings thus indicate that the ZA is characterized by predictable patterns of orthogonal movement with distinct components that can be mapped to specific cytoskeletal effectors, here Myosin II isoforms. This is broadly consistent with recent analyses of cell-cell junctions in Drosophila embryos, which have identified Myosin II-dependent contractile tension at cell-cell junctions [19], [20] and, indeed, patterns of pulsatile contractility that function as morphogenetic drivers [4], [5], [6], [7], [30]. In contrast to Drosophila, however, mammals possess two Myosin II isoforms with significantly different motor and biological properties. Our findings suggest that these functional differences between isoforms extend to their impact upon the dynamic movement of epithelial cell-cell junctions. It should be noted, however, that like genetic mutants, our shRNA approach would potentially have affected many pools of Myosin IIA and IIB within the cells. Recent developments in CALI [20], combined with the analysis of ZA movements that we have now developed, may then provide a useful future approach to investigate how Myosin II isoforms at junctions contribute to cell-cell interactions.

## Materials and Methods

### Cell culture

MCF-7 cells were described previously [16], [32], [33]. Cells were cultured in DMEM complete medium (10% fetal bovine serum, 2 mM glutamine, non essential amino acids, 100 units/ml penicillin, 100 µg/ml streptavidin and 0.01 mg/ml bovine insulin) and propagated as recommended by ATCC.

### shRNA knockdown and lentivirus preparation

E-cadherin shRNA was designed against the ORF of human CDH1 (NM_004360) (5′-GGGTTAAGCACAACAGCAA -3′) and cloned together with a full-length mouse E-cadherin cDNA into a lentivirus expression vector pLL5.0 [26], [34]. In brief, shRNA was cloned downstream of the U6 promoter (HpaI and XhoI) and pLL5.0's EGFP reporter gene was replaced with the RNAi-resistant E-Cad-GFP construct (SacII and SbfI). The E-cadherin-EGFP fusion protein expression was driven by a LTR promoter to facilitate lower expression levels of GFP fusion proteins for imaging. Lentivirus shRNA targeting myosin IIA (MYH9) and myosin IIB (MYH10) were described previously. Lentivirus preparation was performed as described previously [22].

### Antibodies

Primary antibodies were as follows: (1) mouse mAb directed against the extracellular domain of E-cadherin (provided by Dr. M. Wheelock [University of Nebraska, Omaha, NE], with the permission of Dr. M. Takeichi [RIKEN CDB, Kobe, Japan]); (2) rabbit polyclonal antibody (pAb) for non-muscle myosin IIA heavy chain (Covance); (3) rabbit pAb for non-muscle myosin IIB heavy chain (Covance); (4) GFP- rabbit anti-GFP serum (Invitrogen); (5) E-cad- mouse monoclonal IgG2a against cytoplasmic tail (BD Transduction Laboratories); (5) GAPDH-rabbit polyclonal antibody (R&D systems). (6) mouse monoclonal Ab against β-tubulin (Sigma). Secondary antibodies were species-specific antibodies conjugated with AlexaFluor 488 or 594 (Invitrogen) for immunofluorescence, or with horseradish peroxidase (Bio-Rad Laboratories) for immunoblotting.

### Microscopy and Live Cell Imaging

Cells were fixed either with 4% paraformaldehyde in cytoskeleton stabilization buffer (10 mM PIPES pH 6.8, 100 mM KCl, 300 mM sucrose, 2 mM EGTA, 2 mM MgCl_2_) at 22°C for 20 min and subsequently permeabilized with 0.25% Triton-X in PBS for 4 min at room temperature.

Epi-illumination fluorescence microscopy of fixed specimens was performed using an Olympus IX81 microscope equipped ×60, 1.40 NA objectives, driven by Metamorph software (version 7.0, Universal Imaging). Confocal images were captured with a Zeiss 510 Meta laser-scanning confocal microscope, and *z*-stacks were processed with the LSM510 software, or captured with a Zeiss LSM 710 and processed with Zen2009 software.

Live cell imaging was performed on an Olympus spinning disc confocal microscope (IX81 with a disc scanning unit) equipped with a heated chamber (37°C) and humidifier, and images were acquired using a x60, 1.40NA objective. Cells were grown on glass-bottomed dishes (MatTek Corporation, MA, USA) and during imaging, cells were incubated in Hanks balanced salt solution without phenol red supplemented with 10 mM HEPES pH 7.4 containing 5 mM CaCl_2_. For fluorescence time-lapse analysis in MCF-7 cells a full z-stack image was taken every frame (102 seconds) of a defined region in the monolayer with increments of 0.6 µm for the duration of 29 frames (2958 seconds). We generally ran movies for ∼50 min, having found that unidirectional orthogonal movements in a subset of control cells began to decay after 1 hour, suggesting that prolonged imaging may have been associated with phototoxicity. Movies were recorded using Metamorph software and processed with Image J for data analysis and with Imaris imaging software (Bitplane) for presentation purposes.

### Data analysis

#### Data analysis from live cell imaging

A flow chart of the experimental and analysis setup is shown in Fig. 2A. MCF-7 cells were imaged under live conditions and E-Cad-GFP localization in the cells was determined acquiring z-stacks of a section in the monolayer. Sections were chosen according to cells that express soluble mCherrry and at the same time E-Cad-GFP. Movies were taken over a time of 2958 seconds, with 1 frame taken every 102 seconds. Although E-cadherin could be detected along the basolateral to the apical interface between two cells, we could observe regions of enriched E-Cad-GFP signal at the most apical tip of cell-cell contacts. Hence we focused our analysis on the ZA and extracted the corresponding xy-plane for detection of orthogonal movement.

#### Detecting E-cadherin motion

To quantify the motion of E-cadherin between cells in monolayers, an ImageJ (http://rsb.info.nih.gov/ij/) script was created. The input to the script is a tiff stack of images with each slice of the stack representing a time point imaged. The first step was to apply a median filter (radius 0.5 pixels) to reduce noise while preserving edge detail.

To study movement of E-cadherin over the time period of the movie we centered a line perpendicular to a contact and followed the position of E-cadherin from start (t_s_ = 0, green outlined cell) to end of the movie (t_e_ = 2958, magenta outlined cell). As directional movement of E-cadherin at cell contacts was not precisely uniform along the contact area, we found that measuring motion at the peak intensity/midpoint of a contact represented the best trend for the overall movement of E-cadherin. Care was taken that the boundary between the cells was within the extent of the line for all time points (line length = 100 pixels, 0.11 µm/pixel).

For each time point, the script then recorded the position along the line at which the maximum intensity occured. The sequence of positions of such maxima then defined the motion of E-cadherin over time.

#### Decomposing the motion: translation and oscillation

Once the position of the E-cadherin between two cells had been quantified over time, the next step was to consider the components of the orthogonal motion. Firstly, there was the *translation* component. This was obtained by finding the best-fit line to the position data over time. The difference between the values of this line at time t_0_ (experiment start) and t_e_ (end) then gave the *translational distance (µm)* that the boundary membrane moved over the period of observation. The translational distance divided by the time period (t_0 –_ t_e_) of observation, i.e. the *slope* of the best-fit line, gave the *translational velocity (nm/s)* of the membrane.

Subtracting the translational component from the positional data then gave the *oscillatory component* of the motion. This was the back and forth motion of the E-cadherin that is superimposed on the general trend of the motion given by the translation component. To compare experiments, we evaluated the *translational velocities* from 4 different movies with 3–6 contact measurements for each movie.

#### Oscillatory/frequency analysis

In order to better understand the oscillations observed we wished to analyse the frequencies of which the oscillations were composed. Since we are sampling signal amplitudes at discrete time intervals, the discrete Fourier transform (DFT) is the appropriate form of Fourier analysis to decompose the signal into its component frequencies. In practice, we applied the *Fast Fourier Transform* [35] a computationally efficient algorithm for computing the DFT, as implemented in the mathematical software Octave [http://www.octave.org/]. We briefly outline the main properties and limitations of the FFT.

Suppose that experimentally *N* measurements are recorded with a time period of *dt* seconds between measurements. Then the FFT of the signal has components given at each of the frequencies 0, 1/(N*dt), 2/(N*dt), …, (N/2)/(N*dt) = 1/(2*dt), and the power of the signal at each of these frequencies may be calculated by taking the square of the absolute value of the appropriate Fourier component. From this it follows that the lowest (non-zero) frequency that may be measured by the FFT is 1/(N*dt) s^−1^, corresponding to a signal component of period N*dt, i.e. the total time over which measurements were obtained. Similarly, the highest frequency at which power can be measured using the FFT is 1/(2*dt) corresponding to a signal of period 2*dt. This, of course, is the Nyquist limit that requires that samples be taken at a rate of at least twice the period of the highest frequency you wish to measure.

In experiments for which frequency analyses were performed, N = 30 and dt = 102 s, which utilising the spectrum from the FFT gave power P(k) (in arbitary units) at frequencies 0.000327 k, for each k = 1, 2 … 14. (Note 0.000327 = 1/(30*102) and we exclude the power at the Nyquist frequency since it is at the limit of detectability).

To quantify and compare experiments, three metrics were used:

1. *Total power*, the sum of the powers at each frequency in the spectrum is

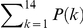

1. *Low frequency power*, the sum of the two lowest frequency powers, those being frequencies at frequencies 0.000327 s^−1^ and 2*0.000327 s^−1^;

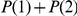

1. *High frequency power*, the sum of the powers at non-low frequencies, given by

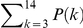



## Supporting Information

Figure S1 **Myosin IIA and IIB knockdown cells expressing E-Cad-GFP.** E-caherin KD/E-Cad-GFP reconstituted cell line was transduced with lentivirus bearing shRNA directed against either Myosin IIA or Myosin IIB. Immunostaining of fixed cells shows depletion of Myosin isoforms from cell-cell contacts and subsequent localization of E-Cad-GFP to junctions. Scale bars = 10 µm.
(TIF)

Figure S2 **Analysis of ZA movement by superimposition of movie frames.** E-cadherin dynamics at cell-cell contacts were visualized using 4D live cell imaging. After acquisition, the time series of a single z-section containing the ZA region was built and projected over time by calculating the standard deviation of pixel intensities values (gray scale data). Superimposed on the standard deviation projection are the t = 0 s (blue), t = 1479 s (green) and t = 2958 s (red) time frames. E-Cad-GFP images are shown from control, Myosin IIA KD and Myosin IIB KD cells. Scale bars = 10 µm.
(TIF)

Figure S3 **Translational components of E-cadherin as best-fit lines of the position data.** Best-fit lines of the translational movements of the E-cadherin from control (A), Myosin IIA KD (B) and Myosin IIB KD (C) cells were calculated from 4 independent movies each and plotted as slopes indicating translational distances at given time. Data are mean_s_ ± SEM (n = 12).
(TIF)

Figure S4 **Representative oscillatory components of the motion of E-cadherin.** The graphs represent calculated oscillatory components of the motion of E-cadherin of control (A), Myosin IIA KD (B) and Myosin IIB KD (C) cells and illustrate the tendency of E-cadherin to deviate around a general trend of motion. The oscillatory component is shown as distance (µm) from the best-fit line. The x-axis corresponds to time (s) and one bar represents oscillatory component at each time frame (102 s/frame).
(TIF)

Movie S1 **4D live cell imaging of E-cadherin in MCF-7 control cells.** The movie shows a z-projection of control MCF-7 cells in a monolayer that express E-Cad-GFP in an E-cadherin knockdown background. 16 z-sections were taken per frame (102 sec) and cells were imaged for 29 frames (2958 sec).
(AVI)

Movie S2 **Orthogonal movements of E-cadherin at the ZA in control cells.** The movie represents movement of cells that express E-Cad-GFP at the ZA from a single z-section of the control movie (Movie S1).
(AVI)

Movie S3 **Orthogonal movements of E-cadherin at the ZA in Myosin IIA KD cells.** The movie shows a z-section of Myosin IIA KD cells that were imaged for movement of E-Cad-GFP at the ZA.
(AVI)

Movie S4 **Orthogonal movements of E-cadherin at the ZA in Myosin IIB KD cells.** The movie illustrates movement of E-Cad-GFP at the ZA of a z-section taken from Myosin IIB KD cells.
(AVI)

## References

[1] Schwartz MA, DeSimone DW (2008). Cell adhesion receptors in mechanotransduction.. Curr Opin Cell Biol.

[2] Smutny M, Yap AS (2010). Neighborly relations: cadherins and mechanotransduction.. J Cell Biol.

[3] Liu Z, Tan JL, Cohen DM, Yang MT, Sniadecki NJ (2010). Mechanical tugging force regulates the size of cell-cell junctions.. Proc Natl Acad Sci U S A.

[4] Martin AC, Gelbart M, Fernandez-Gonzalez R, Kaschube M, Wieschaus EF (2010). Integration of contractile forces during tissue invagination.. J Cell Biol.

[5] Martin AC, Kaschube M, Wieschaus EF (2009). Pulsed contractions of an actin-myosin network drive apical constriction.. Nature.

[6] Sawyer JK, Choi W, Jung KC, He L, Harris NJ (2011). A contractile actomyosin network linked to adherens junctions by Canoe/afadin helps drive convergent extension.. Mol Biol Cell.

[7] Rauzi M, Lenne PF, Lecuit T (2010). Planar polarized actomyosin contractile flows control epithelial junction remodelling.. Nature.

[8] Cavey M, Rauzi M, Lenne PF, Lecuit T (2008). A two-tiered mechanism for stabilization and immobilization of E-cadherin.. Nature.

[9] Blankenship JT, Backovic ST, Sanny JS, Weitz O, Zallen JA (2006). Multicellular rosette formation links planar cell polarity to tissue morphogenesis.. Dev Cell.

[10] Harris TJ, Tepass U (2010). Adherens junctions: from molecules to morphogenesis.. Nat Rev Mol Cell Biol.

[11] Boller K, Vestweber D, Kemler R (1985). Cell-adhesion molecule uvomorulin is localized in the intermediate junctions of adult intestinal epithelial cells.. J Cell Biol.

[12] le Duc Q, Shi Q, Blonk I, Sonnenberg A, Wang N (2010). Vinculin potentiates E-cadherin mechanosensing and is recruited to actin-anchored sites within adherens junctions in a myosin II-dependent manner.. J Cell Biol.

[13] Ladoux B, Anon E, Lambert M, Rabodzey A, Hersen P (2010). Strength dependence of cadherin-mediated adhesions.. Biophys J.

[14] Mege RM, Gavard J, Lambert M (2006). Regulation of cell-cell junctions by the cytoskeleton.. Curr Opin Cell Biol.

[15] Yap AS, Kovacs EM (2003). Direct cadherin-activated cell signaling: a view from the plasma membrane.. J Cell Biol.

[16] Maddugoda MP, Crampton MS, Shewan AM, Yap AS (2007). Myosin VI and vinculin cooperate during the morphogenesis of cadherin cell-cell contacts in mammalian epithelial cells.. J Cell Biol.

[17] Geisbrecht ER, Montell DJ (2002). Myosin VI is required for E-cadherin-mediated border cell migration.. Nat Cell Biol.

[18] Yonemura S, Wada Y, Watanabe T, Nagafuchi A, Shibata M (2010). alpha-Catenin as a tension transducer that induces adherens junction development.. Nat Cell Biol.

[19] Monier B, Pelissier-Monier A, Brand AH, Sanson B (2009). An actomyosin-based barrier inhibits cell mixing at compartmental boundaries in Drosophila embryos.. Nat Cell Biol.

[20] Fernandez-Gonzalez R, Simoes Sde M, Roper JC, Eaton S, Zallen JA (2009). Myosin II dynamics are regulated by tension in intercalating cells.. Dev Cell.

[21] Shewan AM, Maddugoda M, Kraemer A, Stehbens SJ, Verma S (2005). Myosin 2 Is a Key Rho Kinase Target Necessary for the Local Concentration of E-Cadherin at Cell-Cell Contacts.. Mol Biol Cell.

[22] Smutny M, Cox HL, Leerberg JM, Kovacs EM, Conti MA (2010). Myosin II isoforms identify distinct functional modules that support integrity of the epithelial zonula adherens.. Nat Cell Biol.

[23] Conti MA, Even-Ram S, Liu C, Yamada KM, Adelstein RS (2004). Defects in Cell Adhesion and the Visceral Endoderm following Ablation of Nonmuscle Myosin Heavy Chain II-A in Mice.. J Biol Chem.

[24] Vicente-Manzanares M, Ma X, Adelstein RS, Horwitz AR (2009). Non-muscle myosin II takes centre stage in cell adhesion and migration.. Nat Rev Mol Cell Biol.

[25] Bi Y, Shen X, Cong G, Liu X, Chang H (2008). [Establishment of BHK-21 cell lines stably expressing FMDV 3Dpol gene by retroviral-mediated gene transfer technique].. Wei Sheng Wu Xue Bao.

[26] Rubinson DA, Dillon CP, Kwiatkowski AV, Sievers C, Yang L (2003). A lentivirus-based system to functionally silence genes in primary mammalian cells, stem cells and transgenic mice by RNA interference.. Nat Genet.

[27] De la Cruz EM, Ostap EM (2004). Relating biochemistry and function in the myosin superfamily.. Curr Op Cell Biol.

[28] Ishiwata S, Shimamoto Y, Suzuki M (2010). Molecular motors as an auto-oscillator.. HFSP J.

[29] Placais PY, Balland M, Guerin T, Joanny JF, Martin P (2009). Spontaneous oscillations of a minimal actomyosin system under elastic loading.. Phys Rev Lett.

[30] He L, Wang X, Tang HL, Montell DJ (2010). Tissue elongation requires oscillating contractions of a basal actomyosin network.. Nat Cell Biol.

[31] Yap AS, Brieher WM, Pruschy M, Gumbiner BM (1997). Lateral clustering of the adhesive ectodomain: a fundamental determinant of cadherin function.. Current Biology.

[32] Stehbens SJ, Paterson AD, Crampton MS, Shewan AM, Ferguson C (2006). Dynamic microtubules regulate the local concentration of E-cadherin at cell-cell contacts.. J Cell Sci.

[33] Aghamohammadzadeh S, Ayscough KR (2009). Differential requirements for actin during yeast and mammalian endocytosis.. Nat Cell Biol.

[34] Vitriol EA, Uetrecht AC, Shen F, Jacobson K, Bear JE (2007). Enhanced EGFP-chromophore-assisted laser inactivation using deficient cells rescued with functional EGFP-fusion proteins.. Proc Natl Acad Sci U S A.

[35] Brigham EO (2002). Fast Fourier Transform..

