# Supplementary figures and images for: Multicomponent Analysis of Junctional Movements Regulated by Myosin II Isoforms at the Epithelial Zonula Adherens
Source: PLoS One. 2011 Jul 22;6(7):e22458. doi: 10.1371/journal.pone.0022458 (PMC3142163; doi:10.1371/journal.pone.0022458)

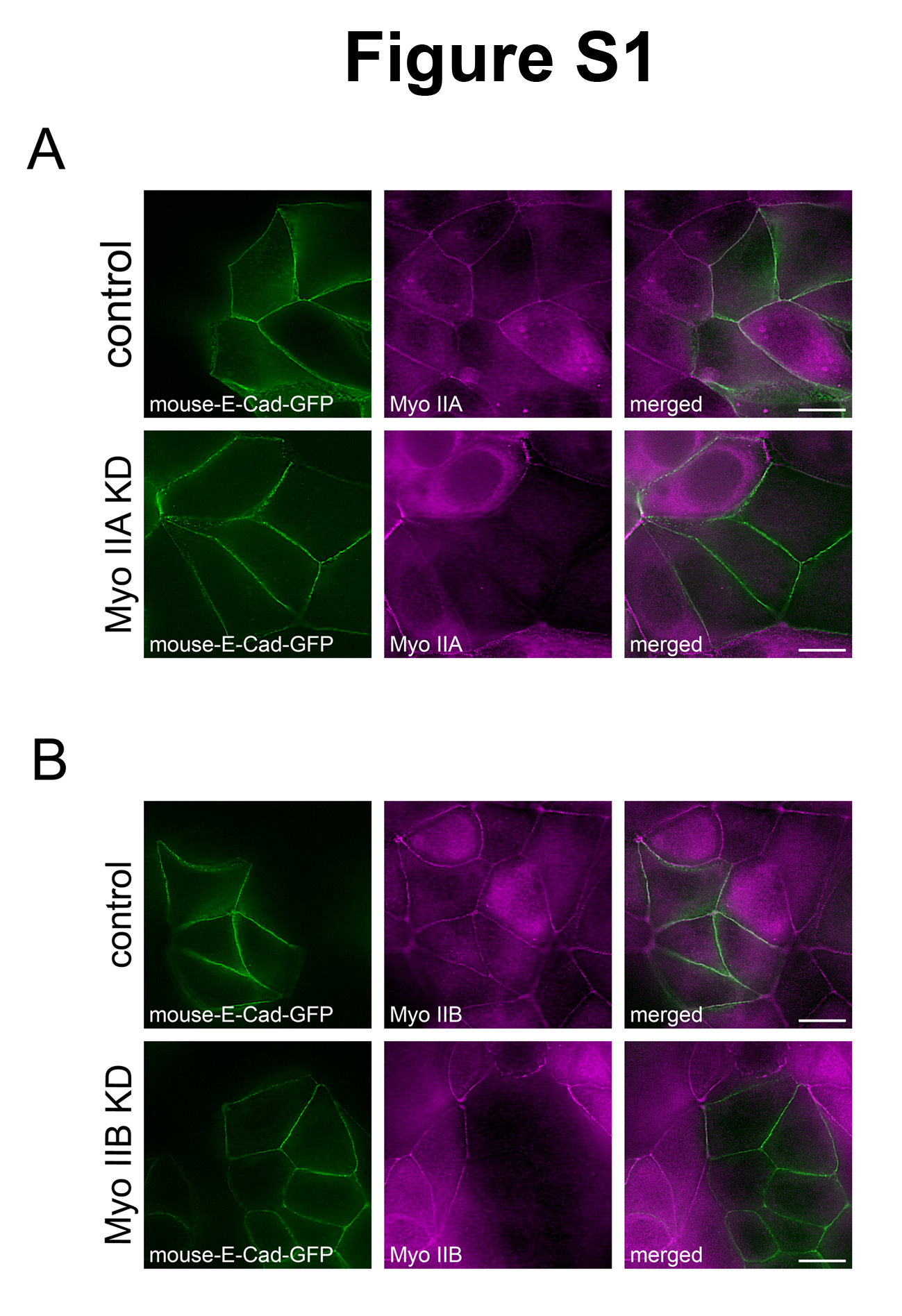

Supplement: Figure S1 — Myosin IIA and IIB knockdown cells expressing E-Cad-GFP. E-caherin KD/E-Cad-GFP reconstituted cell line was transduced with lentivirus bearing shRNA directed against either Myosin IIA or Myosin IIB. Immunostaining of fixed cells shows depletion of Myosin isoforms from cell-cell contacts and subsequent localization of E-Cad-GFP to junctions. Scale bars = 10 µm. (TIF) [file pone.0022458.s001.tif]

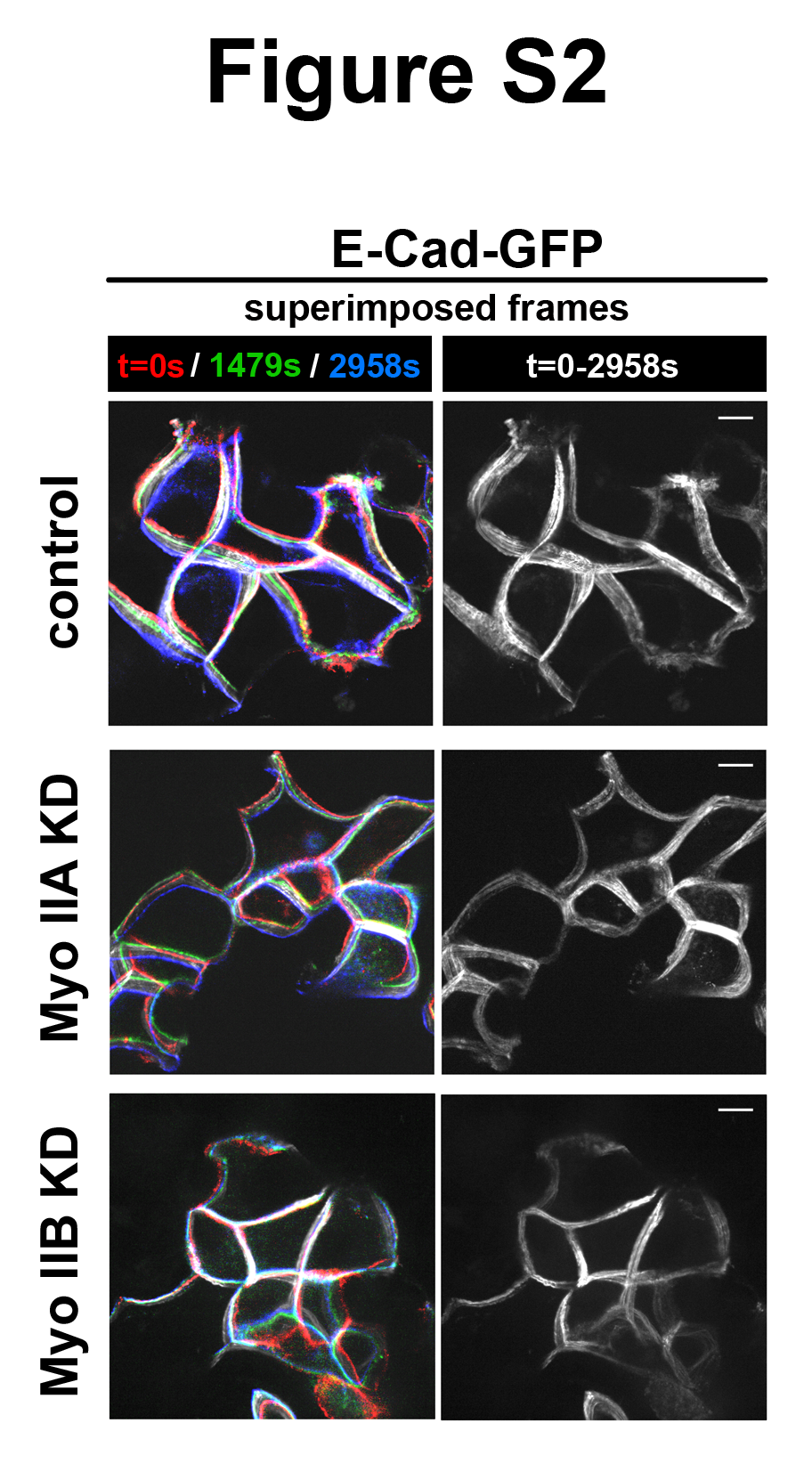

Supplement: Figure S2 — Analysis of ZA movement by superimposition of movie frames. E-cadherin dynamics at cell-cell contacts were visualized using 4D live cell imaging. After acquisition, the time series of a single z-section containing the ZA region was built and projected over time by calculating the standard deviation of pixel intensities values (gray scale data). Superimposed on the standard deviation projection are the t = 0 s (blue), t = 1479 s (green) and t = 2958 s (red) time frames. E-Cad-GFP images are shown from control, Myosin IIA KD and Myosin IIB KD cells. Scale bars = 10 µm. (TIF) [file pone.0022458.s002.tif]

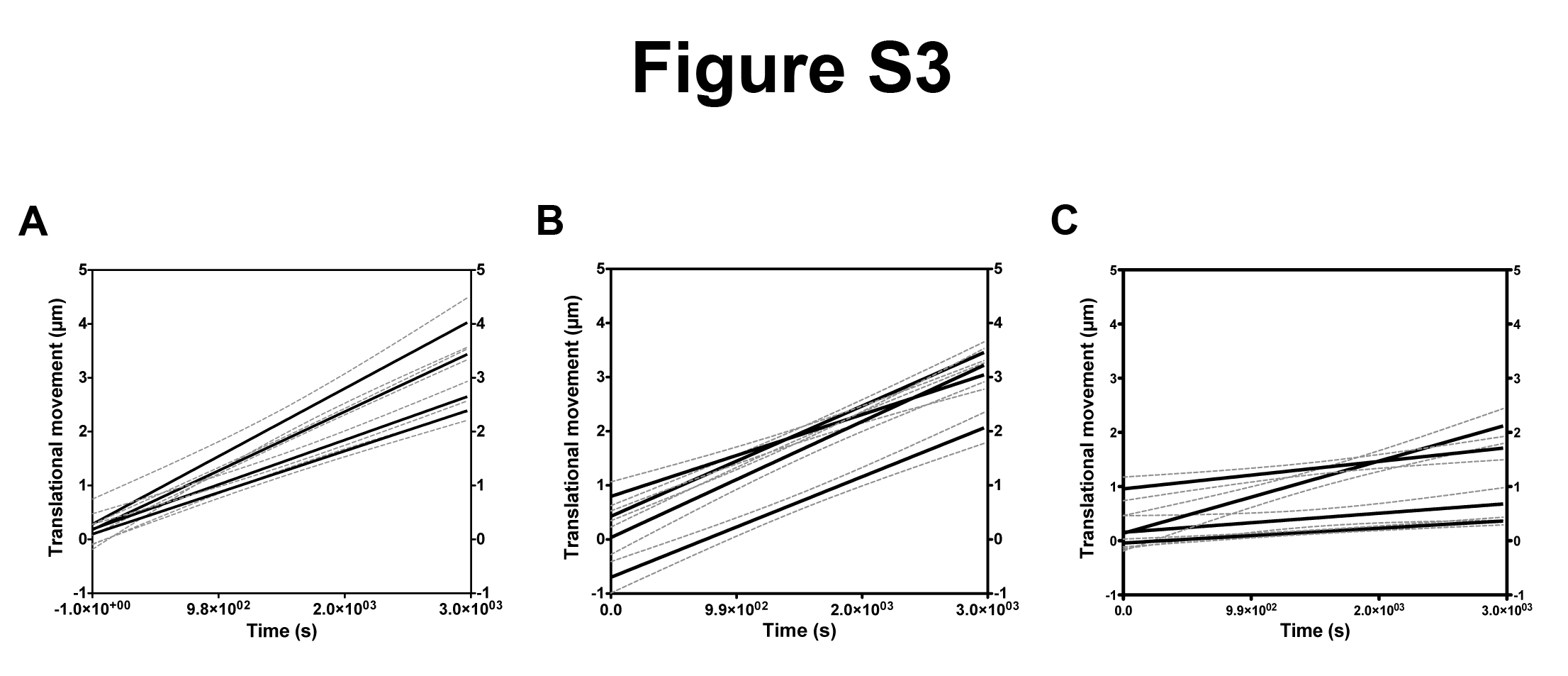

Supplement: Figure S3 — Translational components of E-cadherin as best-fit lines of the position data. Best-fit lines of the translational movements of the E-cadherin from control (A), Myosin IIA KD (B) and Myosin IIB KD (C) cells were calculated from 4 independent movies each and plotted as slopes indicating translational distances at given time. Data are means ± SEM (n = 12). (TIF) [file pone.0022458.s003.tif]

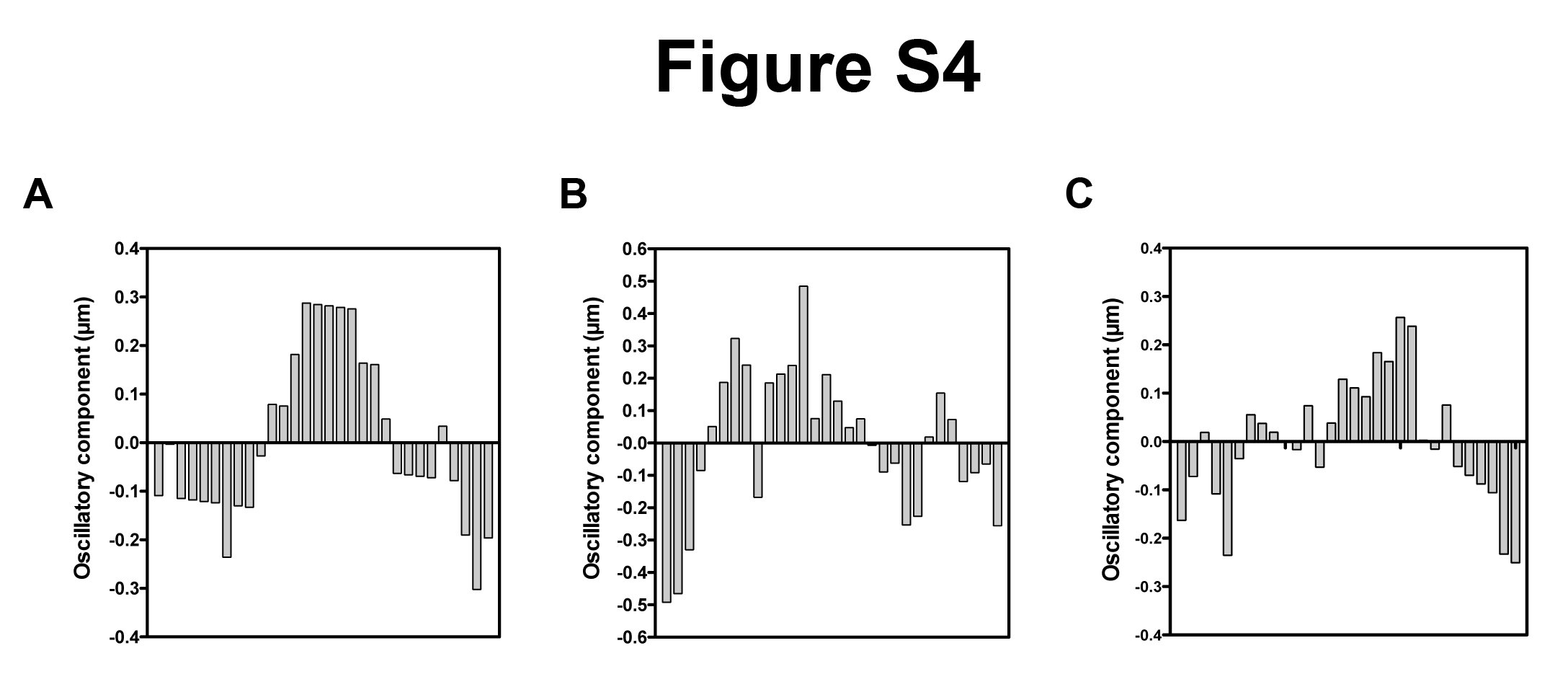

Supplement: Figure S4 — Representative oscillatory components of the motion of E-cadherin. The graphs represent calculated oscillatory components of the motion of E-cadherin of control (A), Myosin IIA KD (B) and Myosin IIB KD (C) cells and illustrate the tendency of E-cadherin to deviate around a general trend of motion. The oscillatory component is shown as distance (µm) from the best-fit line. The x-axis corresponds to time (s) and one bar represents oscillatory component at each time frame (102 s/frame). (TIF) [file pone.0022458.s004.tif]
